# *In Vitro* Activity of Ceftibuten-Avibactam against β-Lactamase-Positive Enterobacterales from the ATLAS Global Surveillance Program

**DOI:** 10.1128/aac.01346-22

**Published:** 2023-01-05

**Authors:** James A. Karlowsky, Meredith A. Hackel, Gregory G. Stone, Daniel F. Sahm

**Affiliations:** a IHMA, Schaumburg, Illinois, USA; b Department of Medical Microbiology and Infectious Diseases, Max Rady College of Medicine, University of Manitoba, Winnipeg, Manitoba, Canada; c Pfizer Inc., Groton, Connecticut, USA

**Keywords:** ceftibuten, avibactam, Enterobacterales, oral therapy, urinary tract infection

## Abstract

Ceftibuten is an established, oral, third-generation cephalosporin in early clinical development in combination with an oral prodrug of avibactam for the treatment of complicated urinary tract infections, including acute pyelonephritis. We evaluated the *in vitro* activity of ceftibuten-avibactam against 1,165 Enterobacterales isolates selected from the 2016–2020 ATLAS global surveillance program based upon their β-lactamase genotype, β-lactam-susceptible phenotype, species identification, and specimen source (95.8% urine). MICs were determined by CLSI broth microdilution. Avibactam was tested at a fixed concentration of 4 μg/mL. Molecular methods were used to identify β-lactamase genes. Ceftibuten-avibactam inhibited 90% (MIC_90_) of ESBL-producing (*n* = 645), KPC-producing (*n* = 60), chromosomal AmpC-positive (*n* = 100), OXA-48-like-producing (*n* = 50), and acquired AmpC-producing (*n* = 110) isolates at concentrations of 0.12, 0.5, 1, 2, and 4 μg/mL, respectively. At concentrations of ≤1 and ≤8 μg/mL, ceftibuten-avibactam inhibited 98.4 and 99.2% of ESBL-positive isolates; 96.7 and 100% of KPC-positive isolates; 91.0 and 99.0% of chromosomal AmpC-positive isolates; 86.0 and 96.0% of OXA-48-like-positive isolates; and 85.5 and 91.8% of acquired AmpC-positive isolates. Against ESBL-producing, KPC-producing, chromosomal AmpC-positive, OXA-48-like-producing, and acquired AmpC-producing isolates, ceftibuten-avibactam was 256-, 128-, >64-, >32-, and > 16-fold more potent than ceftibuten alone. The potency of ceftibuten-avibactam was 4-fold greater than ceftazidime-avibactam against ESBL-producing (ceftibuten-avibactam MIC_90_, 0.12 μg/mL; ceftazidime-avibactam MIC_90_, 0.5 μg/mL) and KPC-producing (0.5 μg/mL; 2 μg/mL) isolates, equivalent to ceftazidime-avibactam (MIC_90_, 2 μg/mL) against OXA-48-like-producing isolates, 2-fold less active than ceftazidime-avibactam (1 μg/mL; 0.5 μg/mL) against chromosomal AmpC-positive isolates, and 4-fold less active than ceftazidime-avibactam (4 μg/mL; 1 μg/mL) against acquired AmpC-producing isolates. Continued development of ceftibuten-avibactam appears justified.

## INTRODUCTION

Avibactam was approved for clinical use by the United States FDA in 2015 and by the European Medicines Agency (EMA) in 2016 as a component of the intravenous only combination ceftazidime-avibactam. Avibactam is a non-β-lactam, diazabicyclooctane inhibitor of Ambler class A β-lactamases, including ESBLs and KPCs, class C (AmpC) β-lactamases, and some class D (OXA-48) β-lactamases. Its ability to reinstate activity to ceftazidime in the majority of Enterobacterales and Pseudomonas aeruginosa isolates that harbor these β-lactamases is now well-established (1). Recently an oral formulation of avibactam (the prodrug ARX-1796) was discovered by Arixa Pharmaceuticals (2). Pfizer purchased Arixa to advance their lead compound (ARX-1796) through clinical trials in combination with ceftibuten, an orally-administered third-generation cephalosporin. Ceftibuten-avibactam (ARX-1796) is in early development for the treatment of complicated urinary tract infections, including acute pyelonephritis.

Oral antimicrobial agents capable of treating complicated urinary tract infections caused by multidrug-resistant (MDR) Enterobacterales carrying ESBLs, acquired AmpCs, and serine carbapenemases comprise a current and important unmet medical need (3, 4). The option to administer oral antibiotics to patients to treat infections with resistant pathogens that currently cannot be treated with oral therapies and require intravenous administration and possibly hospital admission would be an important advance. There are progressively larger numbers of patients who cannot be treated with oral antibiotics and must be treated with an intravenous agent which is also costlier and associated with an additional set of complications. Currently, amoxicillin-clavulanate is the only orally bioavailable β-lactam-β-lactamase inhibitor combination to be FDA-approved for clinical use. It was initially marketed in 1984. Today, its use is fraught with challenges given the increasing numbers of Enterobacterales clinical isolates that carry ESBLs and KPC and OXA-48-like carbapenemases. Clavulanic acid inhibits only a limited number of Ambler class A ESBLs and it is essentially inactive against AmpCs (for which it also acts as an inducer), class A (KPC) and class D (OXA-48-like) carbapenemases, and OXA-1 enzymes commonly associated with ESBL-producing uropathogens. In addition, amoxicillin-clavulanate is generally not ideal for the treatment of urinary tract infections because the concentration of intact clavulanic acid eliminated in the urine is low.

Enterobacterales are the most common pathogens causing both uncomplicated and complicated urinary tract infections. The efficacy of β-lactams for the treatment of Gram-negative infections, including urinary tract infections, is continuously being eroded by the spread of plasmid-mediated ESBLs, AmpCs, and carbapenemases (5, 6). Other currently available oral agents used to treat urinary tract infections also face resistance challenges. Resistance rates among some commonly prescribed empirical agents used to treat patients with urinary tract infections (trimethoprim-sulfamethoxazole, fluoroquinolones) are high (> 20%), while some agents have poor pharmacokinetics (nitrofurantoin), and some have spectrum limitations against common pathogens causing infection (nitrofurantoin, fosfomycin) (3, 7, 8).

In this study, ceftibuten-avibactam and eight comparators were tested against 1,165 clinical isolates of Enterobacterales (mostly urinary tract infection isolates [95.8%; *n* = 1,116]) chosen from isolates submitted to the 2016 to 2020 ATLAS global surveillance program. The isolates tested were pre-selected to include ESBL, KPC, OXA-48-like, chromosomal AmpC, and acquired AmpC enzymes and were intended to determine the ability of avibactam to restore the activity of ceftibuten against a challenge set of commonly encountered β-lactamase-positive Enterobacterales isolates.

## RESULTS

**FIG 1:**
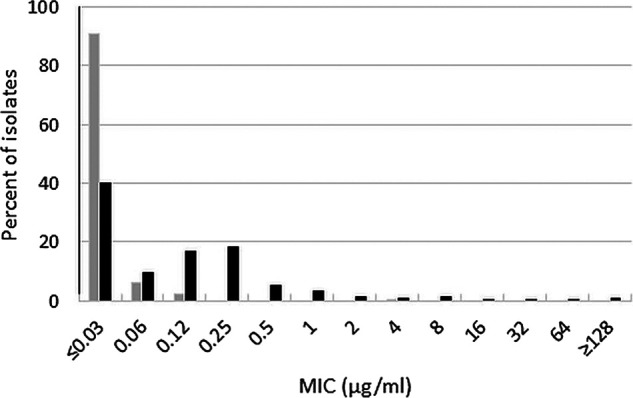
Ceftibuten-avibactam (gray bars) and ceftibuten (black bars) MIC distributions for 200 wild-type Enterobacterales isolates.

Ceftibuten-avibactam inhibited 90% (MIC_90_) of wild-type Enterobacterales isolates at a concentration of ≤0.03 μg/mL; 99.5% of isolates were inhibited at ≤1 μg/mL (EUCAST susceptible breakpoint for ceftibuten for Enterobacterales isolates originating from the urinary tract) (8) and 100% of isolates were inhibited at ≤4 μg/mL (Table 1 and Fig. 1). Ceftibuten alone at a concentration of ≤1 μg/mL, inhibited 93.5% of wild-type isolates compared to 97.5% of isolates at 8 μg/mL (CLSI investigational susceptible MIC breakpoint for ceftibuten for urinary tract isolates of Enterobacterales) (7). Based upon MIC_90_ values, ceftibuten-avibactam was ≥16-fold more potent than ceftibuten alone (MIC_90_, 0.5 μg/mL) and ≥4-fold more potent than ceftazidime-avibactam (MIC_90_, 0.12 μg/mL). All wild-type isolates were ceftazidime-avibactam-susceptible (MIC range, ≤0.03-1 μg/mL). Approximately 70% of wild-type Enterobacterales were susceptible to both levofloxacin (70.5%) and trimethoprim-sulfamethoxazole (69.9%).

**TABLE 1:** *In vitro* activity of ceftibuten-avibactam and comparator agents against wild-type, β-lactamase-positive, and β-lactamase-negative carbapenem-resistant Enterobacterales

		μg/mL			% of isolates susceptible		% of isolates inhibited by ceftibuten-avibactam and ceftibuten at a concn of:			
Phenotype/genotype (*n*)	Antimicrobial agent	MIC_50_	MIC_90_	MIC range	CLSI	EUCAST	≤1 μg/mL	≤2 μg/mL	≤4 μg/mL	≤8 μg/mL
Wild-type^*a*^ (200)	Ceftibuten-avibactam	≤0.03	≤0.03	≤0.03 to 4	NA^*h*^	NA	99.5	99.5	100	100
	Ceftibuten	0.12	0.5	≤0.03 to >64	97.5	93.5	93.5	95.0	96.0	97.5
	Cefepime	≤0.06	0.5	≤0.06 to >32	97.5^*i*^	90.0				
	Cefixime	0.25	2	≤0.12 to >8	88.0	88.0				
	Cefpodoxime	0.5	16	≤0.12 to >32	87.0	84.0				
	Ceftazidime	0.12	1	≤0.03 to >64	95.5	90.5				
	Ceftazidime-avibactam	0.06	0.12	≤0.03 to 1	100	100				
	Levofloxacin	0.12	8	0.008 to >8	70.5	70.5				
	SXT^*g*^	≤0.25	>32	≤0.25 to >32	69.9	69.9				
ESBL-producing^*b*^ (645)	Ceftibuten-avibactam	≤0.03	0.12	≤0.03 to 64	NA	NA	98.4	98.6	99.0	99.2
	Ceftibuten	8	32	≤0.03 to >64	63.6	13.0	13.0	30.5	49.8	63.6
	Cefepime	16	>32	≤0.06 to >32	8.1	3.1				
	Cefixime	>8	>8	≤0.12 to >8	1.7	1.7				
	Cefpodoxime	>32	>32	≤0.12 to >32	0.6	0.5				
	Ceftazidime	16	>64	0.06 to >64	21.6	4.5				
	Ceftazidime-avibactam	0.12	0.5	≤0.03 to >64	99.9	99.9				
	Levofloxacin	>8	>8	0.015 to >8	20.9	20.9				
	SXT	>32	>32	≤0.25 to >32	23.0	23.0				
KPC-producing^*c*^ (60)	Ceftibuten-avibactam	0.12	0.5	≤0.03 to 2	NA^*i*^	NA	96.7	100	100	100
	Ceftibuten	16	64	0.25 to >64	33.3	5.0	5.0	13.3	20.0	33.3
	Cefepime	>32	>32	2 to >32	6.7	0				
	Cefixime	>8	>8	2 to >8	0	0				
	Cefpodoxime	>32	>32	4 to >32	0	0				
	Ceftazidime	64	>64	2 to >64	1.7	0				
	Ceftazidime-avibactam	1	2	≤0.03 to 32	95.0	95.0				
	Levofloxacin	>8	>8	0.06 to >8	5.0	5.0				
	SXT	>32	>32	≤0.25 to >32	18.3	18.3				
OXA-48-like-producing^*d*^ (50)	Ceftibuten-avibactam	0.25	2	≤0.03 to >64	NA	NA	86.0	92.0	94.0	96.0
	Ceftibuten	32	>64	0.12 to >64	26.0	14.0	14.0	16.0	20.0	26.0
	Cefepime	>32	>32	0.12 to >32	10.0	10.0				
	Cefixime	>8	>8	≤0.12 to >8	14.0	14.0				
	Cefpodoxime	>32	>32	1 to >32	6.0	2.0				
	Ceftazidime	>64	>64	0.25 to >64	14.0	12.0				
	Ceftazidime-avibactam	0.5	2	0.06 to 32	94.0	94.0				
	Levofloxacin	>8	>8	0.06 to >8	14.0	14.0				
	SXT	>32	>32	≤0.25 to >32	24.0	24.0				
Chromosomal AmpC-positive^*e*^ (100)	Ceftibuten-avibactam	0.06	1	≤0.03 to 32	NA	NA	91.0	94.0	97.0	99.0
	Ceftibuten	1	>64	≤0.03 to >64	77.0	60.0	60.0	68.0	71.0	77.0
	Cefepime	≤0.06	16	≤0.06 to >32	77.0	70.0				
	Cefixime	2	>8	≤0.12 to >8	38.0	38.0				
	Cefpodoxime	8	>32	0.25 to >32	38.0	27.0				
	Ceftazidime	0.5	64	0.06 to >64	72.0	59.0				
	Ceftazidime-avibactam	0.25	0.5	≤0.03 to 8	100	100				
	Levofloxacin	0.12	8	0.03 to >8	72.0	72.0				
	SXT	≤0.25	>32	≤0.25 to >32	78.0	78.0				
Acquired AmpC-producing^*f*^ (110)	Ceftibuten-avibactam	0.12	4	≤0.03 to >64	NA	NA	85.5	89.1	91.8	91.8
	Ceftibuten	64	>64	0.25 to >64	9.1	0.9	0	2.7	5.5	9.1
	Cefepime	0.5	>32	≤0.06 to >32	59.1	56.4				
	Cefixime	>8	>8	0.25 to >8	1.8	1.8				
	Cefpodoxime	>32	>32	8 to >32	0	0				
	Ceftazidime	32	>64	1 to >64	17.3	0.9				
	Ceftazidime-avibactam	0.12	1	≤0.03 to 4	100	100				
	Levofloxacin	8	>8	0.03 to >8	25.5	25.5				
	SXT	>32	>32	≤0.25 to >32	26.4	26.4				

a Wild-type isolates were defined as isolates of Enterobacterales from species known not to carry an intrinsic, inducible AmpC (Citrobacter amalonaticus, Citrobacter koseri, Escherichia coli, Klebsiella oxytoca, Klebsiella pneumoniae, Klebsiella variicola, Klebsiella oxytoca, Proteus mirabilis, and Raoultella ornithinolytica) that demonstrated an antibiogram of ceftazidime-, cefepime-, aztreonam-, and meropenem-susceptible in initial (previous) testing of isolates with MICs interpreted as susceptible using CLSI MIC breakpoints.

b ESBL-producing isolates were identified by molecular testing (isolates may or may not have carried additional original spectrum TEM or SHV β-lactamases) or by CLSI phenotypic ESBL screening and confirmatory testing.

c KPC-producing isolates were identified by molecular testing (isolates may or may not have carried additional non-carbapenemase β-lactamases).

d OXA-48-producing isolates were identified by molecular testing (isolates may or may not have carried additional non-carbapenemase β-lactamases).

e Chromosomal AmpC-positive isolates were defined as species of Enterobacterales known to carry an intrinsic AmpC (Citrobacter freundii, Enterobacter spp., Klebsiella aerogenes, Morganella morganii, Proteus hauseri, Proteus vulgaris, *Providencia* spp., and Serratia marcescens) irrespective of their antibiogram (isolates may or may not have carried ESBLs but excluded isolates carrying serine carbapenemases and metallo-β-lactamases).

f Acquired AmpC-producing isolates were identified by molecular testing (isolates may or may not have carried ESBLs but excluded isolates carrying serine carbapenemases and metallo-β-lactamases).

g SXT, trimethoprim-sulfamethoxazole.

h NA, not applicable.

i Percentage determined using the CLSI cefepime susceptible-dose dependent breakpoint.

**FIG 2:**
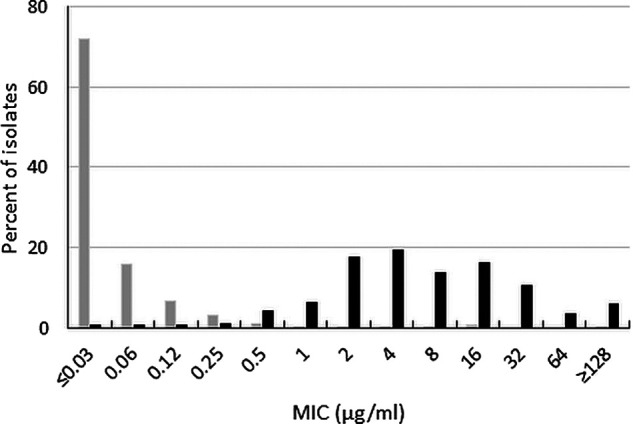
Ceftibuten-avibactam (gray bars) and ceftibuten (black bars) MIC distributions for 645 ESBL-positive Enterobacterales isolates (excludes isolates with chromosomal AmpC and those carrying acquired AmpCs, serine carbapenemases, and metallo-β-lactamases).

Ceftibuten-avibactam inhibited 98.4% of ESBL-producing Enterobacterales at ≤1 μg/mL (MIC_90_, 0.12 μg/mL) and 99.2% of isolates at ≤8 μg/mL (Table 1). A clear separation was observed between ceftibuten-avibactam MICs and MICs to ceftibuten alone (MIC_90_, 32 μg/mL) (Fig. 2); 13.0% and 63.6% of ESBL-producing Enterobacterales were susceptible to ceftibuten alone at ≤1 and ≤8 μg/mL, respectively. Based on MIC_90_s, ceftibuten-avibactam was 256-fold more potent than ceftibuten alone and 4-fold more potent than ceftazidime-avibactam (MIC_90_, 0.5 μg/mL); 99.9% of ESBL-producing isolates were ceftazidime-avibactam-susceptible. Only 20.9% and 23.0% of ESBL-producing isolates were susceptible to levofloxacin and trimethoprim-sulfamethoxazole, respectively.

**FIG 3:**
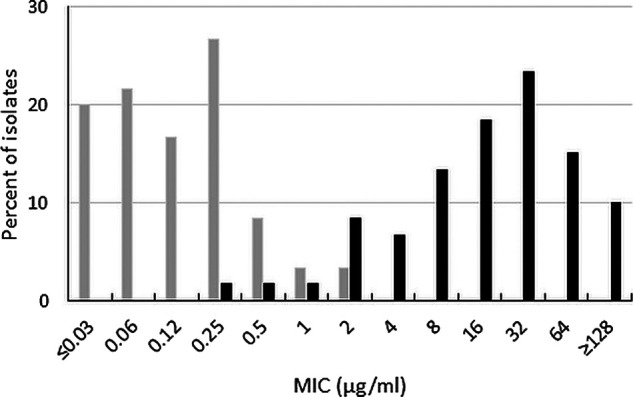
Ceftibuten-avibactam (gray bars) and ceftibuten (black bars) MIC distributions for 60 KPC-positive Enterobacterales (excludes isolates carrying OXA-48-like carbapenemases and metallo-β-lactamases).

Ceftibuten-avibactam inhibited 96.7% of KPC-producing Enterobacterales at ≤1 μg/mL (MIC_90_, 0.5 μg/mL) and 100% of isolates at ≤2 μg/mL (Table 1). Again, a clear separation was observed between ceftibuten-avibactam MICs and MICs to ceftibuten alone (MIC_90_, 64 μg/mL) (Fig. 3); 5.0% and 33.3% of KPC-producing Enterobacterales were susceptible to ceftibuten alone at ≤1 and ≤8 μg/mL, respectively. The addition of avibactam to ceftibuten decreased the MIC_90_ for ceftibuten by 128-fold (from 64 μg/mL to 0.5 μg/mL). Ceftibuten-avibactam was 4-fold more potent than ceftazidime-avibactam (MIC_90_, 2 μg/mL) against KPC-positive isolates; 95.0% of KPC-producing Enterobacterales were ceftazidime-avibactam-susceptible. Only 5.0% and 18.3% of KPC-producing isolates were susceptible to levofloxacin and trimethoprim-sulfamethoxazole, respectively.

**FIG 4:**
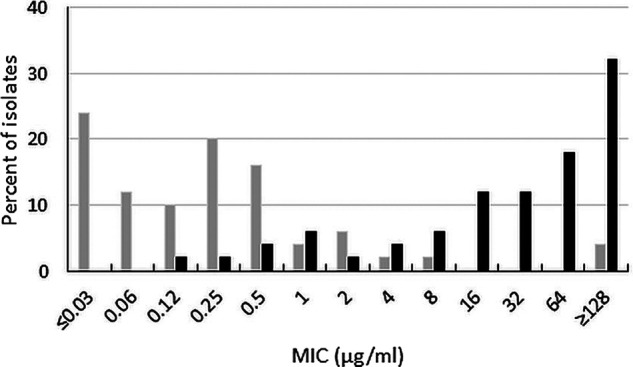
Ceftibuten-avibactam (gray bars) and ceftibuten (black bars) MIC distributions for 50 OXA-48-like-positive Enterobacterales isolates (includes isolates with or without ESBLs and excludes isolates carrying KPCs and metallo-β-lactamases).

Ceftibuten-avibactam inhibited 86.0% of OXA-48-like-producing Enterobacterales at ≤1 μg/mL (MIC_90_, 2 μg/mL) and 96.0% of isolates at ≤8 μg/mL (Table 1). Ceftibuten alone at a concentration of ≤1 μg/mL, inhibited only 14.0% of OXA-48-like-producing isolates and 26.0% of isolates at 8 μg/mL. The addition of avibactam to ceftibuten decreased the MIC_90_ by > 32-fold, from > 64 μg/mL to 2 μg/mL (Fig. 4). Ceftibuten-avibactam and ceftazidime-avibactam had identical MIC_90_ values (2 μg/mL); 94.0% of OXA-48-like-producing Enterobacterales isolates were ceftazidime-avibactam-susceptible. Only 14.0% and 24.0% of OXA-48-like-producing isolates were susceptible to levofloxacin and trimethoprim-sulfamethoxazole, respectively.

**FIG 5:**
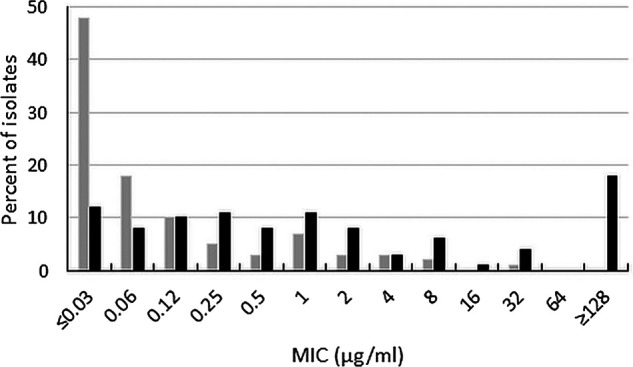
Ceftibuten-avibactam (gray bars) and ceftibuten (black bars) MIC distributions for 100 chromosomal AmpC-producing Enterobacterales isolates (includes isolates with or without ESBLs and excludes isolates carrying serine carbapenemases and metallo-β-lactamases).

Ceftibuten-avibactam inhibited 91.0% of chromosomal AmpC-positive Enterobacterales at 1 μg/mL (MIC_90_, 1 μg/mL) and 99.0% of isolates at ≤8 μg/mL (Table 1). Ceftibuten alone at a concentration of ≤1 μg/mL, inhibited 60.0% of chromosomal AmpC-positive isolates and 77.0% of isolates at 8 μg/mL. The addition of avibactam to ceftibuten decreased the MIC_90_ by > 64-fold, from > 64 μg/mL to 1 μg/mL (Fig. 5). Ceftazidime-avibactam (MIC_90_, 0.5 μg/mL) was 2-fold more active than ceftibuten-avibactam against chromosomal AmpC-positive isolates. All chromosomal AmpC-positive isolates of Enterobacterales were ceftazidime-avibactam-susceptible. Less than 80% of chromosomal AmpC-positive isolates were susceptible to levofloxacin (72.0%) and trimethoprim-sulfamethoxazole (78.0%).

**FIG 6:**
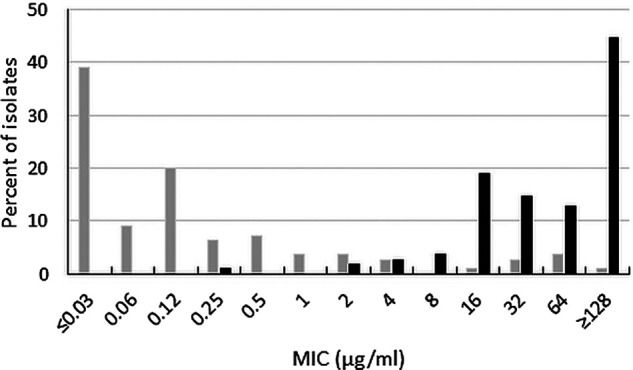
Ceftibuten-avibactam (gray bars) and ceftibuten (black bars) MIC distributions for 110 acquired AmpC-producing Enterobacterales isolates (includes isolates with or without ESBLs and excludes isolates carrying serine carbapenemases and metallo-β-lactamases).

Ceftibuten-avibactam inhibited 85.5% of acquired AmpC-producing Enterobacterales at 1 μg/mL (MIC_90_, 4 μg/mL) and 91.8% of isolates at ≤8 μg/mL (Table 1). Ceftibuten alone at a concentration of ≤1 μg/mL, did not inhibit any isolates with acquired AmpC enzymes and inhibited only 9.1% of isolates at ≤8 μg/mL. The addition of avibactam to ceftibuten decreased the MIC_90_ by > 16-fold, from > 64 μg/mL to 4 μg/mL (Fig. 6). Ceftazidime-avibactam (MIC_90_, 0.5 μg/mL) was 4-fold more active than ceftibuten-avibactam against acquired AmpC-producing isolates; 100% of acquired AmpC-producing isolates of Enterobacterales were ceftazidime-avibactam-susceptible. Less than 30% of acquired AmpC-producing isolates were susceptible to levofloxacin (25.5%) and trimethoprim-sulfamethoxazole (26.4%).

## DISCUSSION

The treatment of urinary tract infections is complicated by the increasing presence of MDR, often β-lactamase-producing Enterobacterales. This trend has resulted in growing numbers of patients with urinary tract infections having to be hospitalized to be treated with intravenous antibiotics because of the failure of oral agents (9–11). ESBL-producing infections are often treated with carbapenems (requiring intravenous therapy) when other non-β-lactams are not an option because of the potential for treatment failure with piperacillin-tazobactam. There is clearly an important unmet need for oral antibiotics that are active against ESBL-producing uropathogens. Oral carbapenems and oral cephalosporin/β-lactamase inhibitor combinations, including ceftibuten-avibactam, are in various stages of clinical development for the treatment of uncomplicated and complicated urinary tract infections caused by MDR and β-lactamase-producing Enterobacterales.

Ceftibuten-avibactam is in early phase development as an oral treatment for complicated urinary tract infections, including acute pyelonephritis. Ceftibuten-avibactam may also have potential as a carbapenem-sparing agent as well as a step-down oral agent from intravenous broad-spectrum empirical or directed parenteral agent therapies (e.g., ceftazidime-avibactam) where Enterobacterales producing serine β-lactamases, including ESBLs or carbapenemases, is known or highly suspected. The ceftibuten-avibactam spectrum of activity includes phenotypes identified by the CDC and WHO as priority pathogens (i.e., carbapenem-resistant and/or third-generation cephalosporin-resistant Enterobacterales) (4). The CDC and WHO both actively promote the development of new agents, particularly oral agents, for outpatient treatment of patients infected with priority pathogens to reduce duration of hospitalization or avoid admission entirely (3, 4).

This study was performed to determine the impact of adding avibactam (4 μg/mL) to ceftibuten to restore the activity of ceftibuten against isolates of Enterobacterales that were ESBL-, AmpC-, and serine carbapenemase-producing. We observed ceftibuten-avibactam MIC_90_ values ranging from 0.12 μg/mL (ESBL-producing isolates) to 4 μg/mL (acquired AmpC-producing isolates) (Table 1 and Fig. 1 to 6) for commonly encountered β-lactamase-positive isolates of Enterobacterales. Ceftibuten-avibactam at a concentration of ≤1 μg/mL inhibited 98.4% of ESBL-producing isolates, 96.7% of KPC-producing isolates, 91.0% of chromosomal AmpC-positive isolates, 86.0% of OXA-48-like -producing isolates, and 85.5% of acquired AmpC-producing isolates. All KPC-producing isolates were inhibited by ceftibuten-avibactam at a concentration of ≤2 μg/mL. Ninety-nine percent of ESBL-producing and chromosomal AmpC-positive isolates were inhibited at ≤8 μg/mL as were 96.0% of OXA-48-like-producing isolates and 91.8% of acquired AmpC-producing isolates.

Only one other, smaller, peer-reviewed study has been published describing the *in vitro* activity of ceftibuten-avibactam. Sader and coworkers reported MIC_50_ and MIC_90_ values, and overall MIC ranges for ceftibuten-avibactam (MIC_50_, ≤0.015 μg/mL; MIC_90_, 0.25 μg/mL; MIC range, ≤0.015 to 0.5 μg/mL; with avibactam tested at a fixed concentration of 4 μg/mL) and ceftibuten (MIC_50_, 0.25 μg/mL; MIC_90_, 0.5 μg/mL; MIC range, 0.06-1 μg/mL) against 13 wild-type isolates (12) that were similar to our observations (Table 1). They also reported ceftibuten-avibactam MIC_50_ and MIC_90_ values against 26 ESBL-producers of 0.03 and 0.12 μg/mL, respectively, and ceftibuten-avibactam MIC ranges of: 0.03 to 0.25 μg/mL for 8 KPC-producers; 1 to 2 μg/mL for 3 isolates with derepressed AmpC; 0.12 to 0.5 μg/mL for 3 isolates with acquired AmpCs; and 0.5 to 4 μg/mL for 2 isolates with OXA-48-like carbapenemases (12). As expected, ceftibuten-avibactam did not exhibit activity against metallo-β-lactamase-producers (*n* = 7) (MIC_50_, > 32 μg/mL) and isolates with porin alterations (*n* = 5) (MIC_50_, 32 μg/mL) (12). Sader et al., surmised that MIC values of ≤4 μg/mL for ceftibuten-avibactam were associated with isolates carrying β-lactamases known to be inhibited by avibactam and that MIC values of > 4 μg/mL were observed for isolates with β-lactamases that were not inhibited by avibactam or had porin alterations (12). These investigators also noted that ceftibuten alone did have *in vitro* activity against some ESBL-positive isolates (46.2% of isolates had ceftibuten MICs ≤1 μg/mL and 61.5% of isolates had MICs ≤4 μg/mL), SME carbapenemases (as does ceftibuten alone), and against some KPC-positive isolates (MICs of 2 to 4 μg/mL) (12). Our data showed similar trends for ESBL- and KPC-producing isolates tested against ceftibuten alone (Table 1).

There are clear differences between current CLSI and EUCAST MIC breakpoints for ceftibuten. CLSI only publishes investigational MIC breakpoints for ceftibuten (susceptible, ≤8 μg/mL; intermediate, 16 μg/mL; and resistant, ≥32 μg/mL) tested against urinary tract isolates of Enterobacterales (7) while EUCAST MIC breakpoints for ceftibuten (susceptible, ≤1 μg/mL and resistant, >1 μg/mL) apply to Enterobacterales infections originating in the urinary tract (8). Clinical and nonclinical studies will be required to determine the dosing of ceftibuten-avibactam and to support the eventual establishment of breakpoints for ceftibuten-avibactam.

Limitations to this study include that isolates were not characterized for non-β-lactamase-mediated resistance mechanisms (e.g., porin mutation/expression and efflux pump expression), which are known to affect the activity of cephalosporins, including ceftibuten, and β-lactam-β-lactamase inhibitor combinations. Moreover, whole genome sequencing was not performed on any isolates suggesting that some isolates may have contained additional resistance mechanisms, besides β-lactamases, that were not directly assessed. Testing amoxicillin-clavulanate as an additional comparator agent may have provided further context to the clinical isolates tested.

We conclude that ceftibuten-avibactam has potential as an oral treatment option for complicated urinary tract infections caused by Class A (ESBL, KPC), C (AmpC), and some Class D (OXA-48-like) β-lactamase-expressing Enterobacterales for which there are currently few oral treatment options available. Continued development of ceftibuten in combination with the oral avibactam prodrug appears justified.

## MATERIALS AND METHODS

### Bacterial isolates.

This study evaluated a subset of 1,165 non-duplicate, clinical isolates of Enterobacterales collected by the annual Antimicrobial Testing Leadership and Surveillance (ATLAS) global surveillance program from 2016 to 2020. Isolates were chosen from the complete collection of ATLAS isolates based upon their β-lactamase genotype, β-lactam-susceptible phenotype, species identification, and specimen source (urine). Urine isolates were chosen for testing (based upon the initial indication for which ceftibuten-avibactam is being developed) except when too few isolates meeting the genotypic, phenotypic, and species identification criteria were available, then isolates from other infection sources were used. Isolates were from (n/percent of total isolates): urinary tract infections (1,116/95.8%), respiratory tract infections (21/1.8%), bloodstream infections (20/1.7%), intraabdominal infections (6/0.5%), and skin and soft tissue infections (2/0.2%). The majority of isolates were collected in (n/percent of total isolates) 2020 (762/65.4%), followed by 2019 (372/31.9%), 2018 (19/1.6%), 2017 (10/0.9%), and 2016 (2/0.2%). Isolates used in this study were provided to ATLAS by medical center laboratories in Africa (*n* = 111), Asia (*n* = 463), Europe (*n* = 199), Latin America (*n* = 169), Middle East (*n* = 61), North America (*n* = 123), and the South Pacific (*n* = 39). All isolates were re-identified at IHMA (Schaumburg, IL; the central testing laboratory for the ATLAS global surveillance program) by matrix assisted laser desorption ionization-time of flight (MALDI-TOF) mass spectrometry (Bruker Daltronics; library version MBT Compass 4.1.90 before December 2019 and version 4.1.100 after December 2019). Speciation of the isolates tested in this study is summarized in Table S1.

For the purpose of this study, wild-type isolates were defined as isolates of Enterobacterales from species known not to carry an intrinsic, inducible AmpC (Citrobacter amalonaticus, Citrobacter koseri, Escherichia coli, Klebsiella oxytoca, Klebsiella pneumoniae, Klebsiella variicola, Klebsiella oxytoca, Proteus mirabilis, and Raoultella ornithinolytica) that demonstrated an antibiogram of ceftazidime-, cefepime-, aztreonam-, and meropenem-susceptible (in previous testing for the ATLAS global surveillance program where isolates were classified as susceptible using CLSI MIC breakpoints). Chromosomal AmpC-positive isolates were defined as species of Enterobacterales known to carry an intrinsic AmpC (Citrobacter freundii, Enterobacter spp., Klebsiella aerogenes, Morganella morganii, Proteus hauseri, Proteus vulgaris, Providencia spp., and Serratia marcescens) irrespective of their antibiogram. As a part of the ATLAS global surveillance program, meropenem-nonsusceptible Enterobacterales isolates, as well as isolates of E. coli, Klebsiella spp. (excluding K. aerogenes), and P. mirabilis testing with aztreonam or ceftazidime MIC values >1 μg/mL, are tested for the presence of genes encoding β-lactamases using published multiplex PCR assays, followed by full-gene DNA sequencing as previously described (13, 14). Qualifying isolates were screened for genes encoding original spectrum β-lactamases (SHV, TEM), ESBLs (CTX-M, GES, PER, SHV, TEM, VEB), serine carbapenemases (GES, KPC, and OXA-48-like), acquired AmpC β-lactamases (ACC, ACT, CMY, DHA, FOX, MIR, and MOX), and metallo-β-lactamases (GIM, IMP, NDM, SPM, and VIM).

### Antimicrobial susceptibility testing.

MICs were determined using the CLSI reference broth microdilution method (15). Broth microdilution panels were prepared at IHMA using cation-adjusted Mueller-Hinton broth (CAMHB) (Becton Dickinson) and stored at −80°C until the day of testing. CAMHB with TES (TREK Diagnostic Systems) was used for inoculum preparation. Tryptic soy agar (TSA) plates containing 5% sheep blood (Remel) were used to subculture isolates.

All antimicrobial agents tested were purchased from commercial sources. MICs for ceftibuten-avibactam and ceftazidime-avibactam were determined at a fixed concentration of 4 μg/mL for avibactam (7, 12). Sader et al., previously determined that the best combination/concentration of ceftibuten with avibactam to separate isolates with β-lactamases inhibited by avibactam from isolates with resistance mechanisms that are not affected by avibactam was doubling dilutions of ceftibuten in combination with avibactam at a fixed concentration of 4 μg/mL (12). MICs for ceftibuten-avibactam were read as the first microdilution panel well with no visible growth following 16 to 20 h of incubation at 35°C in ambient air. Quality control testing was performed each day clinical isolates were tested using E. coli ATCC 25922, P. aeruginosa ATCC 27853, and K. pneumoniae ATCC 700603 (7, 15, 16). MICs were interpreted using CLSI (7) and EUCAST (8) breakpoints. CLSI publishes investigational MIC breakpoints for ceftibuten (susceptible, ≤8 μg/mL; intermediate 16 μg/mL; resistant, ≥32 μg/mL) for testing and reporting of urinary tract isolates only (7). EUCAST publishes MIC breakpoints for ceftibuten (susceptible, ≤1 μg/mL; resistant, >1 μg/mL) for infections originating from the urinary tract (8).
