# Supplementary material for: In Vitro Activity of Ceftibuten-Avibactam against β-Lactamase-Positive Enterobacterales from the ATLAS Global Surveillance Program
Source: Antimicrob Agents Chemother. 2023 Jan 5;67(1):e01346-22. doi: 10.1128/aac.01346-22 (PMC9872606; doi:10.1128/aac.01346-22)
Supplement: Supplemental file 1 — Table S1. Download aac.01346-22-s0001.pdf, PDF file, 0.03 MB [file aac.01346-22-s0001.pdf]

SUPPLEMENTAL TABLE 1 Total isolate counts by species of Enterobacterales

Species of Enterobacterales	Total isolates (% of total isolates)
Citrobacter amalonaticus	1 (0.1)
Citrobacter freundii	13 (1.1)
Citrobacter koseri	11 (0.9)
Enterobacter asburiae	1 (0.1)
Enterobacter bugandensis	2 (0.2)
Enterobacter cloacae	23 (2.0)
Enterobacter cloacae complex	7 (0.6)
Enterobacter kobei	1 (0.1)
Enterobacter spp.	10 (0.9)
Escherichia coli	544 (46.7)
Klebsiella aerogenes	6 (0.5)
Klebsiella oxytoca	16 (1.4)
Klebsiella pneumoniae	400 (34.3)
Klebsiella variicola	9 (0.8)
Morganella morganii	25 (2.1)
Proteus hauseri	2 (0.2)
Proteus mirabilis	47 (4.0)
Proteus vulgaris	10 (0.9)
Providencia rettgeri	13 (1.1)
Providencia spp.	1 (0.1)
Providencia stuartii	9 (0.8)
Raoultella ornithinolytica	1 (0.1)
Serratia marcescens	13 (1.1)
Total	1,165 (100)
